# Does Training Modality Predict Fidelity of an Evidence-based Intervention Delivered in Schools?

**DOI:** 10.1007/s11121-021-01227-6

**Published:** 2021-04-08

**Authors:** Katie Massey Combs, Karen M. Drewelow, Marian Silje Habesland, Marion Amanda Lain, Pamela R. Buckley

**Affiliations:** Institute of Behavioral Science, University of Colorado Boulder, Boulder, CO 80309 USA

**Keywords:** Online training, Fidelity of implementation, Evidence-based intervention

## Abstract

Training prior to implementing evidence-based interventions (EBIs) is essential to reach high levels of fidelity. However, the time and cost of in-person training are often barriers to implementation. Online learning offers a potential solution, though few studies examine the relationship between online training and fidelity of implementation. This study explored whether teachers trained online have similar levels of adherence, dosage, quality of delivery, and student responsiveness compared to teachers trained in-person on the Botvin LifeSkills Training (LST) middle school program, a universal prevention intervention proven to reduce substance use and violence, as part of a national dissemination project. This study involved a sample of 989 LST teachers across 114 school districts, representing 296 schools in 14 states. All teachers were first trained in LST implementation between 2016 and 2019. Hierarchical linear models were used to assess relationships between training modality and the four fidelity outcomes. Online training was associated with lower ratings of quality of delivery compared to in-person training, but no significant associations existed between online training and adherence to the curriculum, dosage, or student responsiveness. Findings from this study generally indicate that online training builds competencies important for school-based EBI implementation, while also highlighting potential shortcomings related to quality of delivery. Ensuring the inclusion of experiential learning activities (e.g., practice delivering content, receiving feedback on delivery) may be key to quality of delivery as online trainings for facilitators of school-based EBIs evolve.

## Introduction

Research has demonstrated that pre-program training for facilitators of evidence-based interventions (EBIs) is essential, though not in itself enough, to reach high levels of fidelity of implementation (Durlak & DuPre, 2008; Dusenbury et al., 2003; Fixsen et al., 2009). Pre-program training is an efficient and effective way to orient facilitators to the background, theory, key practices, and values of an EBI, as well as provide opportunities to practice new methods (Dusenbury et al., 2003; Fixsen et al., 2009). However, pre-program training is just one of multiple considerations critical to implementing an EBI as intended. Aspects such as coaching, assessment, and administrative support have also been identified as key components of strong implementation (Fixsen et al., 2009). Thus, while pre-program training *alone* is an insufficient implementation strategy, it is still a prerequisite for fidelity of implementation, and in turn for EBIs to yield desired outcomes (Durlak & DuPre, 2008; Dusenbury et al., 2003; Fixsen et al., 2009).

Though important to the success of an EBI, the time and costs associated with in-person training can be a barrier to implementation (McMillen et al., 2016). Developers of classroom-based K-12 instructional models of EBIs tend to encourage on-site and in-person training that span 1 or 2 days (National Health Promotion Associates, 2018; PATHS, 2012), which can present economic and pragmatic challenges (e.g., participant travel, schedule coordination, and securing physical space) (Department of Education, 2009). For school-based EBIs, the dynamic nature of staffing in schools further complicates maintaining in-person training for EBI instructors (Becker et al., 2014). Thus, feasible avenues to pre-program training are critical to the scale up and sustainability of school-based EBIs (Drake et al., 2015).

The internet has the potential to facilitate efficient and more affordable sharing of information and learning on a large scale (Calder et al., 2017). Further, the Novel Coronavirus Disease 2019 (COVID-19), declared a pandemic by the World Health Organization on March 11, 2020, has recently (and drastically) shifted the context in which communication, training, and services are delivered. A widely encompassing term, online learning can be defined as educational activities that occur via the internet (Department of Education, 2009). With continued evolution of technology, online learning offers a potential solution to real-world challenges faced in EBI implementation, particularly as modifications are considered to ensure the safe continuity of programming in the context of COVID-19. In addition to economy of scale and public health safety, online training offers advantages over in-person training, including flexibility in where and when courses are taken and consistency in content and delivery (Calder et al., 2017; Department of Education, 2009; McMillen et al., 2016). These pragmatic advantages could be pivotal for EBI scale-up and sustainability.

Despite any hypothesized advantages, modifications to an EBI must not compromise core components that account for intervention efficacy (Elliott & Mihalic, 2004). A logic model is a way to visually represent an EBI’s theory of change and how and why a proven program will work (Moore et al., 2013). While logic models of school-based EBIs may indicate that pre-program training is an essential component (Powers et al., 2010; Moore et al., 2013; Botvin, n.d.), the modality of that training (i.e., in-person or online) is generally not specified. However, the relationship between online training and fidelity of implementation of school-based preventive EBIs is rarely tested. LST, in particular, is theoretically based in teaching youth prevention-related information, promoting anti-drug norms, and fostering personal self-management and general social skills. Training modality is not part of the program’s core causal components (Botvin, n.d.; Botvin & Kantor, 2000); and modifications to LST training should therefore not directly influence outcomes. Thus, this study explored whether teachers trained online have similar levels of fidelity compared to teachers trained in-person on the Botvin LifeSkills Training (LST) middle school program, a universal prevention EBI proven to reduce substance use and violence (Botvin et al., 1995, 2006; Spoth et al., 2002), as part of a national dissemination project conducted between 2016 and 2019.

### Research Comparing Online Versus In-Person Learning

Though few studies have compared differences in fidelity of implementation for EBI facilitators who are trained online versus in-person, a body of research exists that examines online learning in various fields for an array of outcomes. Several systematic reviews (Calder et al., 2017; Rohwer et al., 2017) and meta-analyses (Cook et al., 2008; Department of Education, 2009) have compared online to no training, online to hybrid learning, and online to in-person training for topics, including education and evidence-based practices for healthcare professionals. Such studies have included a wide range of professionals and examined a range of outcomes, including knowledge, attitudes, skills, and behavior.

Specifically, considerable research has been conducted on training clinicians and health professionals to implement evidence-based practices (Calder et al., 2017; Herschell et al., 2010; Hubley et al., 2015; McMillen et al., 2016). For example, two systematic reviews and a meta-analysis compared differences in knowledge, attitudes, behavior, and skills between healthcare professionals (e.g., doctors, nurses, dentists, mental health counselors) trained online, in-person, or through blended/hybrid trainings. Cook et al. (2008) conducted a meta-analysis with 201 studies involving healthcare professionals in trainings on a wide variety of medical topics, including evidence-based medicine, communication, and biostatistics; Rohwer et al. (2017) reviewed 24 studies with medical professionals in trainings on evidence-based healthcare interventions, and Calder et al. (2017) included 16 studies with addiction counselors and mental health clinicians who received training on evidence-based approaches including cognitive behavioral therapy, motivational interviewing, and medication assisted treatment. In each of these studies, findings indicated that health professionals who attended online formats had greater knowledge and skills, and more positive attitudes towards the topic compared to those who received no training. Across the three studies, however, differences when comparing in-person versus online trainings were often small, leading authors to conclude that online training and traditional in-person training methods showed similar effectiveness. For example, Cook et al. (2008) found that online trainings outperformed no training (*d* = 1.00 for knowledge outcomes, 0.85 for skills, and 0.82 for learner behaviors), while online compared to in-person forms of training showed no difference in skills or behavioral outcomes and a small difference favoring online formats in knowledge (*d* = 0.12). In comparing blended approaches, Rohwer et al. (2017) found that hybrid/blended learning outperformed both in-person-only and online-only modalities.

Studies undertaken in the field of education have drawn parallel conclusions. For example, the Department of Education conducted a meta-analysis of 51 studies comparing in-person learning to online learning, as well as to blended forms of learning across a range of settings including education for K-12, college and graduate students, and professional development of educators. Though this meta-analysis found insufficient evidence to produce effect sizes or draw conclusions regarding the effectiveness of online learning for K-12 students, it found that outcomes (e.g., standardized tests, researcher developed assessments of knowledge, supervisor’s rating of job performance) for adult learners in online classes exceeded those of learners in traditional face-to-face classes (*g* = 0.24). Blended or hybrid models further exceeded purely in-person modalities (*g* = 0.35) (Department of Education, 2009).

Overall, the literature suggests that online training *for adults* is comparable to in-person training (Calder et al., 2017; Cook et al., 2008; Department of Education, 2009), while online training consistently surpasses comparison conditions of reading written manuals or no training (Calder et al., 2017; Cook et al., 2008; Department of Education, 2009; Rowher et al., 2017). Still, several limitations in this body of research have been noted. First is that studies encompass a wide variety of online trainings, making it difficult to decipher whether differences are due to online learning itself or to the quality of a given online module (Calder et al., 2017; Cook et al., 2008; Department of Education, 2009). Additionally, studies included in systematic reviews and meta-analyses often had limitations such as small sample sizes, failure to report attrition rates, and potential bias from researchers’ dual roles as experimenters and instructors (Department of Education, 2009; Rohwer et al., 2017). Despite these limitations, the effectiveness of online learning appears wide-ranging across different types of content and adult learners.

In reference to the current study, it is critical to acknowledge that the various learners (e.g., medical professionals versus graduate students) and types of online learning (e.g., distance learning for a college or graduate class versus in-service professional development) represented in the aforementioned systematic reviews are different in context and in bodies of literature from training teachers to implement an EBI with fidelity, which research has documented is notoriously challenging (Dusenbury et al., 2003; Fixsen et al., 2009; McMillen et al., 2016). That is, distance learning for a graduate student is distinct from training healthcare providers to implement evidence-based practices, and both are distinct from training teachers to deliver an evidence-based prevention program to youth. Whether training modality predicts fidelity of an EBI delivered in schools is largely unknown, which is a gap that our study serves to address.

### Online Versus In-Person Training and Fidelity of Implementation

While few studies have examined differences in fidelity of implementation for teachers implementing school-based EBIs, one notable contribution is a randomized control trial that compared fidelity of implementation of a classroom-based EBI between teachers who received online training and teachers who received no training (Drake et al., 2015). The online training had moderate, positive effects on teachers’ self-reported dosage compared to no training (*d* = 0.31). Only one study has compared fidelity of implementation for teachers in online trainings versus in-person trainings (Becker et al., 2014). The authors conducted a correlational study comparing online training to in-person training among teachers in a wait-list control group from three elementary schools in which all teachers received in-person classroom coaching after training. Coaching included support with preparing materials and classrooms, instructional modeling, observations, and technical assistance. The online training group had similar levels of fidelity of implementation compared to the in-person training group (Becker et al., 2014). This study was limited in that it included a small sample of urban elementary school teachers and was part of a rigorously controlled study (e.g., teachers completed the online training at school with research assistants to provide support, and received in-person coaching) (Becker et al., 2014). Finally, previous studies did not account for various socio-ecological factors that influence the implementation of school-based EBIs (Domitrovich et al., 2008; Durlak & DuPre, 2008).

### Current Study

The present study examined the relationship between training modality and fidelity of implementation of the Botvin LifeSkills Training (LST) middle school EBI. Given the potential advantages of online training, we posed the following research question: *Do teachers trained online have similar levels of adherence, dosage, quality of delivery, and student responsiveness compared to teachers trained in-person?* This study builds upon previous research by including a large sample of teachers in urban/suburban and rural settings, controlling for factors associated with fidelity of implementation, and comparing differences between teachers in online trainings and in-person trainings on four domains of fidelity of implementation (Dusenbury et al., 2003).

## Methods

We used process evaluation data of teachers’ first year of implementation in a project supporting LST dissemination across 14 states between academic years 2016–2017 and 2018–2019. The sample included 989 teachers across 296 schools and 114 school districts. A university institutional review board confirmed that no ethical approval was required due to the exclusive use of retrospective data that were part of routine process evaluation.

### LifeSkills Training Dissemination Project

The LifeSkills Training (LST) middle school program is a classroom-based intervention implemented with students in grades 6–8 or 7–9 and incorporates personal self-management skills (e.g., self-esteem, problem solving, coping with stress, and anxiety), social skills (e.g., communication, building relationships, and assertiveness), and drug resistance skills (e.g., consequences of drug use, refusal skills). A total of 30 LST sessions are divided into three levels, taught in sequence over 3 years. Following the prescribed LST model, teachers delivered 15 sessions to 6th/7th grade students, 10 sessions to 7th/8th grade students, and 5 sessions to 8th/9th grade students. Lessons were generally facilitated by classroom teachers using a range of teaching techniques, including didactic instruction, discussion, demonstration, and behavior skill rehearsals. LST lessons were meant to be taught one to five times per week until all core lessons were delivered. Optional violence prevention lessons were included in the curriculum as well. In general, the program developer recommends the curriculum be delivered in a traditional classroom, with up to 25 students, in roughly 45-min class periods, and by means of a teacher’s manual and consumable student guides.

In lieu of monetary incentives, participating schools were provided with LST curriculum materials, training, technical assistance, and process evaluation activities at no cost. This included annual visits by dissemination project coordinators trained in the LST middle school model to meet with LST teachers, observers, and school administration to discuss implementation progress and problems. Additionally, phone-based technical assistance was provided to teachers upon request or as needed.

One to five local observers were hired per school district to collect process evaluation data for the dissemination project. Based on a structured job description, observers were recommended by school district personnel, with the explicit guidance that observers should not be school staff or familiar with the teachers implementing to reduce bias. The observers were required to attend a pre-program LST training, as well as a standardized 60-min session detailing the processes and procedures with the fidelity measurement tools. In the majority of cases, observers were unaware of the training modality (online or in-person) of the teachers that they observed, though it was possible for observers to know if they were in the same in-person training or had extensive familiarity with the school. Observers were instructed to observe each Level 1 teacher four times (27% of Level 1 core sessions), each Level 2 teacher three times (30% of Level 2 core sessions), and each Level 3 teacher two times (40% of Level 3 core sessions). In their first year of implementation, teachers were observed between two and seven times (*m* = 2.7, *SD* = 1.2), depending on which level(s) of LST they taught in their first year delivering LST.

During the classroom observations, observers completed a checklist and survey assessing fidelity of implementation. Observers were instructed to not provide feedback to teachers on their lessons. However, *teachers* could request feedback from their observations through the dissemination project coordinators. Additionally, a formal report was provided at the end of each year summarizing process evaluation data collected from their respective school districts. During annual site visits, project coordinators conducted joint classroom observations with the local observers to validate the accuracy of the information recorded. Across the three-year project, on average, observers and coordinators had agreement on 90.9% of responses. This observation measure has been used in numerous LST evaluations and has demonstrated inter-rater reliability consistently above 0.80 (Botvin et al., 1995; Mihalic et al., 2008).

### Training

Per the LST logic model, both online trainings and in-person trainings were facilitated by National Health Promotion Associates Inc. (NHPA) certified LST trainers (Botvin, n.d.). Reasons teachers attended an online training included scheduling conflicts with the districts’ in-person training, time of hire, or inability to travel to an in-person training. Online trainings followed an agenda designed to cover similar material and objectives as the in-person trainings. Teachers could complete the online training in any environment with access to a computer with internet, phone, and program materials. The training included five sessions, totaling six training hours; the first and last sessions were 90-min live trainer-led presentations, and the three middle sessions were 60-min each with self-paced material. The two live trainer-led sessions were synchronous-only (not recorded for later access). Participants were required to be in front of a computer screen and connected to audio via a phone or their computer. The trainer guided participants through presentation slides featuring content on program background, fundamentals, fidelity guidelines, and modifications. During the trainer-led sessions, participants had the opportunity to ask the trainer questions and respond to questions using a chat or “raise hand” feature. Self-paced sessions involved participants working independently to review the program’s scope and sequence, instructional design, theoretical foundation, and interactive teaching skills, and to deconstruct a lesson. The self-paced session information was covered through pre-recorded presentations and demonstration videos modeling the interactive teaching skills. The second live session involved the trainer reviewing and discussing participants’ lesson deconstruction assignments. Teachers in the online training received a certificate of completion verifying that they attended the two live sessions and completed activities in the self-paced sessions; 100% of teachers in the online trainings (*n* = 107) for this study received a certificate of completion.

The in-person training spanned either one or two 6-hour days, depending on the number of participants or the district’s scheduling parameters. All content covered in the online training was included in the in-person trainings; however, differences existed in the style of delivery (i.e., less didactic with more experiential activities). For example, unlike the online trainings that presented interactive teaching skills through video, these skills were covered through live trainer modeling and peer-to-peer practice and feedback during in-person trainings. In 2-day in-person trainings (and some 1-day in-person trainings if time allowed), participants practiced teaching a select portion of the LST curriculum. As with the online training, 100% of in-person trained teachers (*n* = 882) completed their training and received a certificate of completion.

### Sample

A total of 127 districts participated in the dissemination project, which included 371 schools and 1608 teachers. Because this project was a continuation of a multi-year, multi-cohort dissemination project, a portion of districts (and thus teachers) had previously been trained in LST. To ensure that the online training variable indicated teachers who attended online training *only*, the sample was limited to teachers who trained in LST for the first time between 2016 and 2019. Additionally, because some online trained teachers attended in-person refresher trainings after their first year of implementation, only data from teachers’ first year of implementation, after the initial pre-program training, were analyzed. Thus, this analytic sample included teachers who were first trained in LST implementation by an NHPA trainer between the 2016–2019 project years, examining only their first year of implementation. This resulted in a total of 989 teachers (107 in online trainings, 882 in in-person trainings) across 114 school districts, representing 296 schools. Though health teachers comprised the largest portion of LST teachers involved in this project, the program’s curricular placement and LST teachers’ area of expertise varied by school, including areas such as social studies, science, language arts, and advisory.

Schools had an average of 6.7 teachers implementing LST across the 3-year program; however, approximately three-quarters (75.7%) had three or fewer teachers. Districts had an average of 17.2 teachers implementing LST, with nearly one-half (47.4%) having three or fewer teachers. Given the small sample of teachers within schools, and because schools within a district shared characteristics, administration, and an LST coordinator, clustering of teachers was accounted for within districts, rather than schools. On average, districts consisted of 3.2 schools (*SD* = 5.08); 47.4% of sites included just one school, 28.0% included two or three schools, and 24.6% included four or more schools. Just over one-half (53.5%) of the 114 districts were in rural areas, and 46.5% were in suburban/urban areas. On average, school district student bodies were 59.1% white (range: 0% to 99% White) and 40.9% were youth of color.

### Measures

#### Dependent Variables

Outcomes were four fidelity of implementation domains: adherence, dosage, quality of delivery, and student responsiveness (Durlak & DuPre, 2008; Dusenbury et al., 2003), measured through classroom observations. Average scores were constructed for each teacher’s first year of implementation for the four dependent variables. *Adherence* was measured through a checklist created by the developer of LST (National Health Promotion Associates, 2013), which indicated the percentage of core activities and lesson points in the manual that an instructor covered. *Dosage* included the average number of minutes spent on an LST lesson (Botvin et al., 2018; Domitrovich et al., 2015). Each lesson was designed to last approximately 45 min. Observers were trained to report the precise lesson start time, lesson end time, and note any interruptions to instruction (e.g., announcements, fire drills, redirecting student misbehavior) and subtract this time from the total number of minutes. Therefore, the dosage measure represents the time specifically dedicated to lesson delivery. *Quality of delivery* was measured through seven items assessing the instructor’s delivery of lessons. Items asked about the teacher’s knowledge of the program, enthusiasm, poise and confidence, rapport and communication, classroom management, ability to address questions, and overall quality of the lesson. Response options were on a Likert scale (1 = Poor to 5 = Excellent) and had strong internal reliability (Chronbach’s alpha = 0.97). The seven items were averaged to create a mean quality of delivery score. *Student responsiveness* was assessed by three items asking how well students understood, participated in, and responded to the lesson. Response options were on a Likert scale (1 = Poor to 5 = Excellent) and had high internal reliability (Chronbach’s alpha = 0.94).

#### Independent Variables

The primary independent variable was training modality: online training (1) or in-person training (0). Controls included two teacher-level (level 1) and eight district-level (level 2) variables. The level 1 control variables included average class size and proportion of lessons in which an observer noted a problem. Class size was the average number of students in teachers’ observed classes. For proportion of lessons with problems, each observed lesson was coded as yes (1) or no (0) for experiencing one or more challenges related to lack of materials, shortage of time (e.g., interruptions due to fire drills), student misbehavior, or inadequate facilities (e.g., lesson was taught in a gymnasium with no desks). Then, the percentage of observed lessons in which a problem was observed was calculated.

The eight district-level controls were percentage of students identifying as white, locale, number of schools in the district participating in the dissemination project, teacher support of LST, administrative support of LST, and district-level means of the three level 1 variables. The district’s grant application, collected as part of enrollment in the project, provided locale (i.e., rural or urban/suburban). During program implementation, each district provided the percentage of students identifying as white or as a youth of color who received LST. At the end of their first implementation cycle, teachers were asked how much they agreed with the following statements: *I am in favor of having the LST program in my school* and *school administrators were supportive of the LST program* (1 = Strongly disagree to 5 = Strongly agree). Scores for the respective teacher and administrator support items were aggregated across each district. Lastly, district-level means were calculated for the three level 1 variables to control for potential contextual effects of these teacher-level variables. Specifically, district-level means of level 1 predictors were proportion of teachers trained online, average class size, and average percent of lessons with problems.

### Analysis

Two-level hierarchical linear models (HLM) were estimated in HLM 7.01 to account for nesting of teachers within districts for the four fidelity of implementation outcomes. Prior to analyzing data in HLM, associations between all variables were examined in SPSS to check for collinearity issues. The strongest correlation among the level 1 variables was between “proportion of lessons with problems” and “student responsiveness” (*r* = 0.47), and the strongest correlation among level 2 variables was between “teacher support of the program” and “administrative support of the program” (*r* = 0.41), thereby ruling out multi-collinearity concerns.

Four separate models were run, one for each outcome (i.e., adherence, dosage, quality of delivery, student responsiveness), and each model included the same set of theoretically pre-determined predictors with teacher variables at level 1 and district variables at level 2. Continuous predictors were centered at the grand mean; binary predictors remained uncentered. Intraclass correlations (ICC) were estimated for each outcome using an empty model (i.e., random intercept only). The ICCs showed that 17.8% of variability in adherence, 38.7% in dosage, 18.9% in quality, and 19.9% in responsiveness were due to district-level differences, indicating the need for multi-level models. For each model, we reported pseudo-*R*^2^ (measure of the proportion of variance explained in the dependent variable) and deviance statistics (measure of model fit). Though there is no simple parallel for *R*^2^ to that obtained in a standard ordinary least squares regression, several proposed measures for pseudo-*R*^2^ exist. We used the formula recommended by Snijders and Bosker (1999), which distinguishes the proportion of variance accounted for in the teacher-level outcome by the teacher-level predictors from the variance accounted for in the district-level means by the district-level predictors (Snijders & Bosker, 1999). Though deviance statistics (− 2*log likelihood) do not have meaning alone, they can be used to compare nested models, and smaller values generally indicate stronger model fit.

## Results

Table 1 displays descriptive statistics for each dependent variable, as well as teacher-level (i.e., level 1) independent variables and district-level (level 2) independent variables. On average, teachers covered 75.2% of curriculum content in observed lessons and spent 44.2 minutes on LST lessons. The average quality of delivery score was 4.2, and average student responsiveness score was 4.2, representing an average rating of “very good.” For each model, deviance statistics decreased from the unconditional to the final models. Pseudo-*R*^2^ values ranged from 2.5% for dosage to 27.1% for student responsiveness. As shown in Table 2, online training was associated with lower ratings of quality of delivery compared to in-person training. Though each had a negative direction, no significant association existed between online training and adherence, dosage, or student responsiveness.

**Table 1:** Descriptive statistics

	M (SD)/%	Range	Reporter/Data source
Dependent variables, *N* = 989			
Adherence	75.2 (21.2)	2.5–100.0	Observer
Dosage	44.2 (13.3)	15.3–123.3	Observer
Quality of delivery (alpha = .97)	4.2 (0.8)	1.1–5.0	Observer
Student responsiveness (alpha = .94)	4.2 (0.7)	1.0–5.0	Observer
Level 1 (teacher-level) variables, *N* = 989			
Online trained	10.80%	-	Administrative
Average class size	22.1 (6.3)	4.0–61.3	Observer
Proportion of lessons with problems	32.2% (38.1)	0.0–100%	Observer
Level 2 (school district-level) variables, *N* = 114			
Number of schools within school district	3.0 (5.0)	1.0–48.0	Administrative
Rural district	53.50%	-	District
Districts’ average proportion of white students	59.1% (30.0)	0.2–98.5%	District
Teacher support	4.1 (0.6)	2.0–5.0	Teacher
Administrative support	4.3 (0.4)	3.1–5.0	Teacher
District average proportion of teachers trained online	12.1% (23.3)	0.0–100.0%	Administrative
District average class size	21.5 (4.2)	10.5–33.3	Observer
District average: proportion of lessons with problems	30.9% (22.2)	0.0–92.0%	Observer

**Table 2:** Hierarchical linear models assessing associations between online training and fidelity of implementation

			Adherence			Dosage			Quality			Responsiveness		
		Variable	B	SE	*t*-ratio	B	SE	*t*-ratio	B	SE	*t*-ratio	B	SE	*t*-ratio
Level 2 – School district		Intercept	77.82***	1.87	41.61	44.15***	1.16	38.06	4.35***	0.06	70.04	4.28***	0.06	74.81
		No. of schools	− 0.19*	0.09	− 2.11	0.12	0.14	0.83	− 0.01***	0.00	− 4.23	− 0.01***	0.00	− 5.57
		Rural	− 2.73	2.55	− 1.07	− 0.36	1.76	− 0.21	− 0.01	0.10	− 0.09	0.07	0.08	0.82
		% white students	0.10*	0.04	2.44	0.04	0.03	1.39	0.01***	0.00	3.52	0.01	0.00	4.22
		Teacher support	0.22	2.89	0.08	− 1.17	1.47	− 0.79	0.10	0.09	1.10	0.07	0.08	0.97
		Admin support	− 5.68	3.32	− 1.71	− 3.41	2.11	− 1.62	− 0.03	0.12	− 0.25	0.01***	0.11	0.13
		% online trained	− 5.91	6.10	− 0.97	− 0.09	2.70	0.03	− 0.12	0.18	− 0.68	0.01	0.14	0.07
		District class size	0.17	0.27	0.63	0.35	0.28	1.24	0.00	0.01	0.26	0.00	0.01	0.46
		% lessons with problem-district	− 0.66	5.49	− 0.12	− 8.90	4.59	− 1.94	0.00	0.20	0.01	0.02	0.15	0.15
Level 1-Teacher		Online trained	− 2.30	3.10	− 0.74	− 2.04	1.50	− 1.36	− 0.17*	0.08	− 2.01	− 0.10	0.06	− 1.54
		Class size	0.16	0.11	1.47	0.00	0.07	− 0.07	0.00	0.01	0.60	0.00	0.00	0.20
		% lessons with problem	− 8.11***	2.05	− 3.95	− 0.58	1.24	− 0.47	− 0.86***	0.08	− 10.19	− 0.78***	0.07	− 10.95
Psuedo-*R*^*2*^			0.040			0.025			0.246		0.271			

The four dependent variables had different scale ranges, and thus, unstandardized coefficients (B) can vary dramatically across models. The* t*-ratio is a standardized coefficient that allows for comparison of the strength of association of a predictor across models.

Models showed decreases in deviance statistics from unconditional to the full models presented here: Adherence unconditional model deviance = 8755, adherence full model deviance = 8694; Dosage unconditional model deviance = 7411, dosage full model deviance = 7384; Quality unconditional model deviance = 2211, quality full model deviance = 2019; Responsiveness unconditional model deviance = 1953; responsiveness full model deviance = 1749.

Ranges of the dependent variables were adherence: 2.5–100.0, dosage: 15.3–123.3, quality: 1.1–5.0, responsiveness: 1.0–5.0

*p < .05; **p < .01; ***p < .001

## Discussion

This study expands upon the limited research concerning online training for facilitators of school-based EBIs (see Drake et al., 2015 and Becker et al., 2014) by including a large sample of urban/suburban and rural teachers across 14 states and controlling for socio-ecological factors that have been found to influence the fidelity of implementation of school-based EBIs. We found that online training built the competencies of teachers in our sample to implement the LST program with fidelity. Specifically, there were no statistically significant differences in training modality on three of four domains typically used to measure fidelity of implementation (Durlak & DuPre, 2008; Dusenbury et al., 2003): adherence, dosage, or student responsiveness. Our findings support the potential of online training to ensure classroom-based EBIs are implemented with fidelity, which is important given the recent shift to online trainings with COVID-19 (and whether this will continue is unknown), coupled with the advantages of online trainings to potentially provide more efficient, affordable sharing of information on a large scale.

While promising, findings also highlight shortcomings of online pre-program training for facilitators of EBIs; specifically, online training was associated with lower ratings of quality of delivery compared to in-person training. Overall, measures of fidelity of implementation were high in this sample, including those for quality of delivery. A threshold for “quality of delivery” has not been established, and since student outcomes were not assessed, whether this reduction in quality of delivery would translate to poorer student outcomes is unknown. However, studies suggest that while quality of delivery is not as commonly assessed as measures of dosage or adherence, it is likely even more important for maintaining student outcomes (Durlak & DuPre, 2008; Humphrey et al., 2018). Thus, the reduction in quality of delivery for teachers trained online observed in this sample is noteworthy and highlights where attention should be focused as programs and developers consider online pre-program training formats.

The LST program relies heavily on peer-to-peer learning and interactive teaching techniques. Online training is often more independent and less interactive, with fewer opportunities for teachers to practice and receive feedback using these techniques. For example, in this online training, participants did not practice or receive feedback on delivering a lesson. In McMillen et al. (2016) study assessing clinicians’ experiences in a web-based training for EBIs, they noted that these components (practice and feedback) were necessary for course completion and active engagement. Further, research largely supports the notion that pre-program training is most effective when it is structured to include modeling, practice or role playing, and performance feedback in a safe and supportive training environment (Durlak & DuPre, 2008; Fixsen et al., 2009; Sterling-Turner et al., 2001). Indeed, according to adult learning theory and professional development models, these active and experiential forms of learning, (e.g., practice, coaching, classroom application) are essential to the transfer of knowledge and skills into regular instructional practices (Conlan et al., 2003; Joyce & Showers, 2002).

Quality of delivery of a classroom-based EBI, therefore, may be enhanced if online trainings ensure the integration of experiential learning and interactive methods. As online trainings evolve, training providers should ensure proper time and means (e.g., instructions, platform) for participants to practice curriculum delivery and receive feedback from the trainer and from peers. Specifically, practicing delivery of lessons provides an opportunity for hands-on experience with the content with a trainer available for guidance, as well as a preview of lesson content for fellow learners. Additionally, feedback enhances the learners’ understanding of best practices and promotes learner-to-learner and learner-to-trainer dialogue about implementation, both of which can translate to higher quality of delivery in the classroom.

Of note, the model for dosage (i.e., average length of lessons in minutes) did not perform well in our analyses, given that the theoretically pre-determined variables associated with fidelity of implementation included in our model did not explain much variation. We maintained dosage as a study outcome, however, because lesson length has been used to assess dosage throughout the literature (Botvin et al., 2018; Domitrovich et al., 2015), and dosage is one of the most common measures of fidelity of implementation cited in the literature (Durlak & DuPre, 2008). For the context of this project, lesson length was constrained by the length of the class period, which was determined by the school or district. It is possible that our measure of lesson length was inherently tied to decisions made at the administrative level and thus not affected by typical factors associated with teachers’ fidelity of implementation; this idea is supported by the higher intra-class correlations (ICCs)—a statistic used to describe the average correlation between individuals in the same cluster or group, in this case how strongly associated teachers were in the same school district—for dosage (38.7%) compared to the ICCs of the other three outcomes in our study (each of which were below 20%). Though teachers could have carried lessons over to the next class, or utilized more classes, if they were limited by a short class period, such realities could not be reflected in our measure of dosage (or “average length of lesson”). Future research should carefully assess and consider whether the measure of dosage used in the present study is an accurate reflection of the amount of curriculum (or intervention) received by students participating in a school-based EBI.

Interpretation of these results, as well as those from the other known study comparing online training to in-person training for facilitators of a school-based EBI (Becker et al., 2014), must consider that some level of continuing support was provided throughout implementation of the EBIs. The in-person training and online training groups in Becker et al. (2014) included in-person classroom coaching after initial, pre-program trainings. While pre-program training introduces knowledge, theory, and skills to implement an EBI, coaching is on-the-job advice that is specific and ongoing for an individual teacher (Fixsen et al., 2009). Though the current dissemination project did not provide the same intensive and universal in-person coaching and consultation as did Becker et al. (2014), the project did include phone-based and on-site technical assistance to teachers upon request. This assistance was generally provided after teachers had begun delivering lessons and had identified specific training areas of interest. In addition, this project provided ongoing process evaluations, another implementation component that is considered critical for fidelity (Fixsen et al., 2009). Research is clear that pre-program training alone is insufficient for fidelity of implementation of an EBI, and it is important to emphasize that these results (as well as Becker et al.’s) do not reflect online training versus in-person training *only*, but rather online or in-person training *plus* some level of continuing support. Within this context, online training can be an appropriate method to provide pre-program training to facilitators of a school-based EBI and is especially suitable when in-person training is not feasible. This conclusion comes with caution; however, that increased attention is needed to ensure that methods of practicing and receiving feedback on facilitators’ skills to deliver the curriculum content are incorporated into online pre-program trainings.

### Limitations

Selection bias is a major limitation in this study, inhibiting our ability to draw casual inferences. In-person training was the default training for this dissemination project, and online training served as a “backup” when that was not possible. This resulted in the online training group being relatively small compared to the in-person training group. Further, the reason for some teachers’ participation in online trainings may have related to their motivation for implementing the EBI or characteristics of their environment (e.g., turnover). In addition, this study did not differentiate between 1-day and 2-day in-person trainings. Because 2-day in-person trainings included more teacher-practice, it is possible that the results of 1-day trainings more closely resemble those of online trainings. Moreover, while findings from this study shed light on how online training compares to in-person training for teachers implementing an EBI, they are not necessarily generalizable to *all* online trainings, especially online trainings using only asynchronous formats, rather than the mix of synchronous and asynchronous sessions in this study. With the COVID-19 pandemic, EBIs quickly transitioned to online training and thus the format of online trainings are rapidly evolving; the online training examined in this study may not reflect the exact formats or versions that are currently in widespread use.

Measurement reliability is another limitation of this study. Though observer-reported measures are advantageous and are often considered more objective compared to teacher self-reported measures or those collected at the end of implementation (Durlak, 2015), inconsistencies and personal biases may have existed in observers’ assessments of fidelity of implementation. While school districts were asked to recommend observers who were not school district staff, it is possible that observers had a relationship with teachers they observed since they lived in proximal communities, and since they could have attended the same pre-program training. Though we do not expect that these situations occurred frequently, it could contribute to inflated ratings on fidelity measures. To reduce inconsistency and guard against bias, all observers were trained in the LST model and in assessing fidelity of implementation of the model. In addition, observers were advised to refrain from providing teachers with any feedback.

Finally, this study focused specifically on fidelity of implementation outcomes (e.g., we did not assess teachers’ ratings of the training or its adequacy) and was limited to observed variables available in our dataset. As mentioned previously, the measure of dosage (i.e., average length of lessons in minutes) was imperfect and may not accurately reflect the amount of curriculum that students received. Additionally, though the models included controls associated with fidelity of implementation throughout the literature, several key variables were unavailable, namely, teachers’ years of experience, age, and perceptions of their capacity to implement an EBI (i.e., self-efficacy) (Mihalic et al., 2008; Pas et al., 2015; Payne & Eckert, 2010; Wang et al., 2017). Finally, researchers were unable to assess differences in teachers’ use of technical assistance (i.e., phone-based or on-site coaching) between in-person and online-trained groups.

## Conclusion

Online training offers multiple potential benefits to implementers of EBIs, such as efficiency, lower cost (compared to in-person), consistency in content and delivery, and greater access. Findings indicated that online training builds competencies important to implementing an EBI with fidelity as teachers in the online and in-person trainings had similar scores on adherence, dosage, and student responsiveness—all noteworthy results. Attention should be given to potential shortcomings of online training regarding quality of delivery. Focusing on quality of delivery by integrating experiential learning, practicing delivery of content, and receiving feedback are important as online training for the implementation of EBIs evolve.

The COVID-19 pandemic rapidly changed the landscape of how education and training occur. Certainly, perceptions of online training, and online trainings themselves will transform, especially given that COVID-19 forced most pre-program EBI trainings to quickly navigate to an online forum. At present, whether online training will become the new business-as-usual model for preparing facilitators to deliver school-based EBIs is still largely unknown. This study notes some of the positives, as well as areas of attention (i.e., quality of delivery), for practitioners weighing the pros and cons of online pre-program training. As such, further investigation on this topic is vital in monitoring the fidelity of implementation of classroom-based instructional EBIs.
