# Correction to: Does Training Modality Predict Fidelity of an Evidence-based Intervention Delivered in Schools?

**DOI:** 10.1007/s11121-021-01250-7

**Published:** 2021-05-06

**Authors:** Katie Massey Combs, Karen M. Drewelow, Marian Silje Habesland, Marion Amanda Lain, Pamela R. Buckley

**Affiliations:** Institute of Behavioral Science, University of Colorado Boulder, Boulder, CO 80309 USA

## Correction to: Prevention Science https://doi.org/10.1007/s11121-021-01227-6

The article "Does Training Modality Predict Fidelity of an Evidence‑based Intervention Delivered in Schools?", written by Katie Massey Combs, Karen M. Drewelow, Marian Silje Habesland, Marion Amanda Lain, and Pamela R. Buckley, was originally published electronically on the publisher’s internet portal on 08 April 2021 without open access. With the author(s)’ decision to opt for Open Choice the copyright of the article changed on 21 April 2021 to © The Author(s) 2021 and the article is forthwith distributed under a Creative Commons Attribution 4.0 International License, which permits use, sharing, adaptation, distribution and reproduction in any medium or format, as long as you give appropriate credit to the original author(s) and the source, provide a link to the Creative Commons licence, and indicate if changes were made. The images or other third party material in this article are included in the article’s Creative Commons licence, unless indicated otherwise in a credit line to the material. If material is not included in the article’s Creative Commons licence and your intended use is not permitted by statutory regulation or exceeds the permitted use, you will need to obtain permission directly from the copyright holder. To view a copy of this licence, visit http://creativecommons.org/licenses/by/4.0/.

The original article has been corrected.

